# Pulmonary involvement from animal toxins: the cellular mechanisms

**DOI:** 10.1590/1678-9199-JVATITD-2023-0026

**Published:** 2023-09-18

**Authors:** Suthimon Thumtecho, Suchai Suteparuk, Visith Sitprija

**Affiliations:** 1 Division of Toxicology, Department of Medicine, Chulalongkorn University, King Chulalongkorn Memorial Hospital, the Thai Red Cross Society, Bangkok, Thailand.; 2 Queen Saovabha Memorial Institute and King Chulalongkorn Memorial Hospital, the Thai Red Cross Society, Bangkok, Thailand.

**Keywords:** lung injury, animal, toxin, cellular mechanism, pulmonary edema, pulmonary hemorrhage

## Abstract

Venomous animals and their venom have always been of human interest because, despite species differences, coevolution has made them capable of targeting key physiological components of our bodies. Respiratory failure from lung injury is one of the serious consequences of envenomation, and the underlying mechanisms are rarely discussed. This review aims to demonstrate how toxins affect the pulmonary system through various biological pathways. Herein, we propose the common underlying cellular mechanisms of toxin-induced lung injury: interference with normal cell function and integrity, disruption of normal vascular function, and provocation of excessive inflammation. Viperid snakebites are the leading cause of envenomation-induced lung injury, followed by other terrestrial venomous animals such as scorpions, spiders, and centipedes. Marine species, particularly jellyfish, can also inflict such injury. Common pulmonary manifestations include pulmonary edema, pulmonary hemorrhage, and exudative infiltration. Severe envenomation can result in acute respiratory distress syndrome. Pulmonary involvement suggests severe envenomation, thus recognizing these mechanisms and manifestations can aid physicians in providing appropriate treatment.

## Backgrounds

Humans have long been fascinated by venomous animals and their venom. Animals from both terrestrial and aquatic habitats, such as wasps, bees, spiders, scorpions, and snakes, as well as fish, sea urchins, cone snails, cnidarians, and annelids, all create venom for various purposes such as predation, defense, and competition reduction [ 1]. Venom are abundant natural sources of biogenic amines, proteins, and peptides [ 2, 3]. Due to the high metabolic expense of venom generation, a wide range of potent and selective toxins have been developed [ 4] to specifically target key physiological components of the target species [ 1]. Even though venomous animals normally do not hunt humans, coevolution has equipped venom with the ability to attack human physiological structures, usually, those engaged in crucial regulatory processes or bioactivities such as cell membranes, ion channels, and receptors [ 5]. Despite being much smaller in size, even a small amount of their poisons can result in catastrophic injury or even death.

One of the serious consequences of animal toxins is respiratory failure from neuromuscular dysfunction or lung injury, whose underlying mechanisms are rarely discussed. This review demonstrates how specific toxin components can cause lung injury through various biological pathways.

### Mechanisms for pulmonary involvement by animal toxins

As early as the Anthozoa phylogeny (sea anemones and corals), some animals harness the capability of producing toxins [ 6]. Over generations, significant proportions of the common (ancestral) or lineage-specific genes and gene families [ 5, 6] responsible for toxin production affect the fundamental physiology of the target organisms, including humans.

Because toxins are readily distributed into the systemic circulation and various organs through the vascular and lymphatic systems once released and injected, one of the serious consequences of animal toxins is respiratory failure resulting from lung injury [ 7]. With the high blood flow and extensive vascular bed, the pulmonary system serves as a major target for toxins. In addition to altering the physiology and architecture of gas exchange barriers, the diverse mixture of toxins also dysregulated the host immune response [ 8].

**Figure 1.**
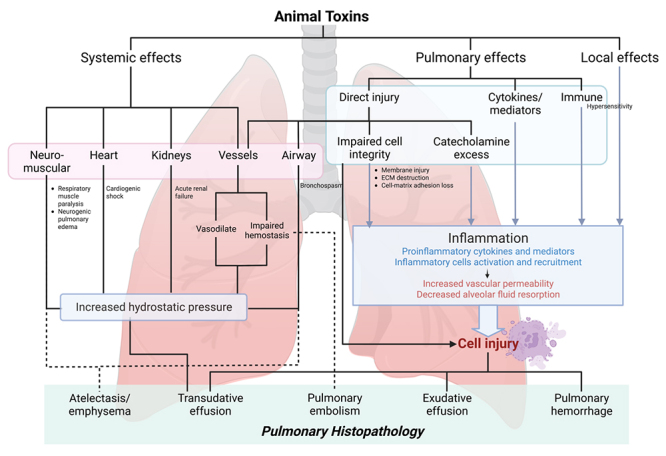
Pathophysiology of animal toxin-induced lung injury and the resulting pulmonary histopathology. Three main toxin-induced lung injuries include damages to the pulmonary system, non-pulmonary systems, and damages at the site of toxin entry. Toxins can exert systemic effects, leading to elevated pulmonary hydrostatic pressure and subsequent development of pulmonary edema. Additionally, toxins can directly harm the lungs or contribute to secondary damage through local or systemic inflammation. The observed histopathology is influenced by the underlying pathophysiological mechanism. ECM: extra-articular matrix.

Envenomation can cause lung injury through three broad categories of toxin-induced end-organ damage: pulmonary, non-pulmonary, and local site of toxin entry ( Figure 1). Many toxins exert systemic effects that indirectly raise pulmonary hydrostatic pressure and result in pulmonary edema. Several case reports and reviews have highlighted cardiotoxicity leading to cardiodepression, myocarditis, and myocardial infarction [ 9- 13]; nephrotoxicity leading to volume overload [ 14, 15]; neurotoxicity leading to respiratory muscle paralysis and ventilatory failure [ 16, 17], and neurogenic pulmonary edema [ 18]. These causes of pulmonary injuries are mentioned elsewhere. Several toxins directly damage the lungs by increasing airway resistance, which leads to atelectasis or emphysema [ 19]. On a microscopic level, many toxins cause pulmonary edema (transudate or exudates), hemorrhage, or embolism through numerous cellular mechanisms, most of which are followed by lung inflammation whose pathophysiologic derangements resemble those of acute lung injury or acute respiratory distress syndrome (ARDS) [ 20, 21]. 

**Figure 2.**
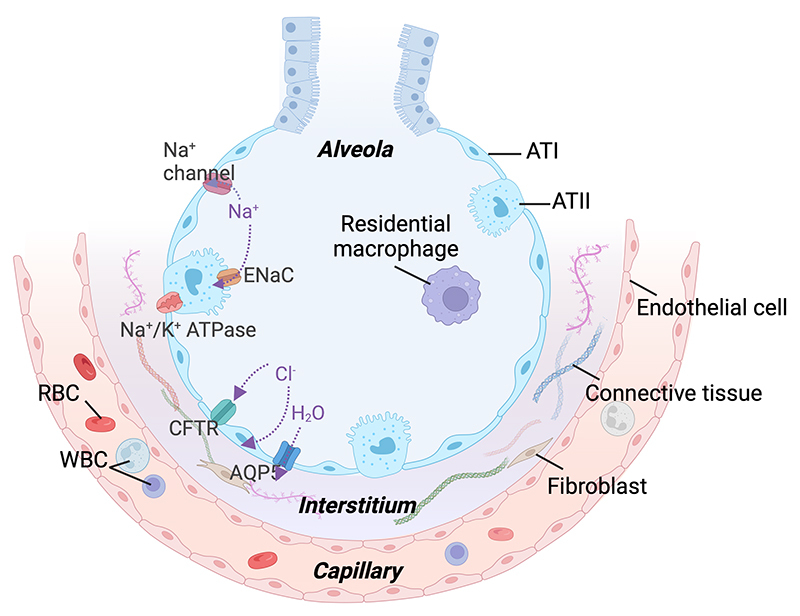
Normal cellular structures and important ion channels of the alveolus. The alveolar unit consists of a single-layer endothelium composed of ATI and ATII cells, creating a selective barrier for fluids and solutes. The alveolar fluid is primarily regulated through the absorption of sodium via ENaC and basolateral Na^+^/K^+^ ATPase pumps, as well as water through AQP5 channels, while the electrochemical and osmotic gradients are maintained by the CFTR chloride channel. Additionally, the alveolar unit includes structural extracellular matrix components and immune cells such as alveolar macrophages, neutrophils, and monocyte. AT: alveolar type; AQP5: aquaporin 5; CFTR: cystic #brosis transmembrane conductance regulator; ENaC: epithelial sodium channel; Na^+^/K^+^ ATPase: sodium/potassium ATPase pump; RBC: red blood cell; WBC: white blood cell; Na^+^: sodium, K^+^: potassium, Cl^-^: chloride, H_2_O: water.

**Figure 3.**
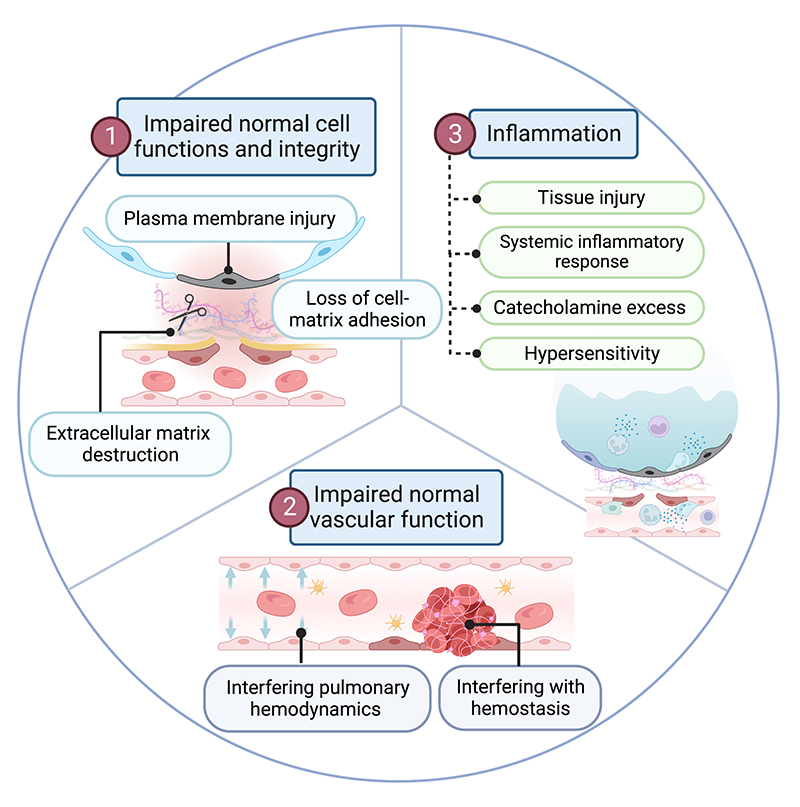
Proposed cellular mechanisms of animal toxin-induced lung injury. The figure illustrates the common mechanisms that collectively contribute to the development of lung injury from animal toxin exposure. The mechanisms include interference with essential cellular functions and integrity necessary for cell survival, disruption of normal vascular function through diverse mechanisms of action, and induction of excessive inflammation, which can indirectly contribute to cellular damage.

The alveolar unit, a critical part of the pulmonary system, is lined by a single-layer endothelium of alveolar type II (ATII) and flat alveolar type I (ATI) cells to form a selective barrier to fluids and solutes. To maximize gas exchange, these cells remove excess airspace fluid by creating an osmotic gradient through the absorption of sodium by apical epithelial sodium channels (ENaC) and basolateral Na^+^/K^+^ ATPase pumps, and water by aquaporin channels (AQP) such as AQP5. The electrochemical and osmotic gradients are also maintained by a chloride channel called cystic fibrosis transmembrane conductance regulator (CFTR) [ 21, 22]. The alveolar unit also consists of essential structural extracellular matrix (ECM) components such as basement membrane (BM) and interstitial connective tissues [ 23], and immune cells such as alveolar macrophages, neutrophils, and monocytes [ 20] ( Figure 2). Understanding the structural basis of the pulmonary system provides insight into lung pathologies induced by toxins [ 21]. In this article, we propose the common underlying cellular mechanisms by which animal toxins can cause lung injury ( Figure 3).

## Impaired normal cell functions and integrity 

***Plasma membrane injury***

Cell membrane integrity is crucial for maintaining cellular compartments and ionic homeostasis. Disruptions in this balance can induce secondary inflammation and cell death [ 24, 25]. Membrane damage can be caused by enzyme digestion or the insertion of positively charged amphipathic peptides (pore formation) [ 26] found in many venomous species, including cnidarians, fish, insects, arachnids, and snakes [ 27]. The common membrane toxins include:

a) Phospholipase A_2_ (PLA_2_): 

PLA_2_ is widely distributed among venomous animals [ 28]. This fundamental and prevalent enzyme toxin mimics the mammalian housekeeping PLA_2_, hydrolyzing the glycerophospholipids in cytosolic organelles and plasma membranes, leading to non-specific membrane disruption, loss of cytosolic calcium homeostasis, and eventual cell degeneration. Because of its non-specific membrane affinity, PLA_2_ produces various effects including neurotoxicity, myotoxicity, cardiotoxicity, and local tissue damage [ 29]. 

Snake venom PLA_2_

The basic PLA_2_ from Russell's viper ( *Daboia russelli*) (VRV-PL-VIIIa) can induce pulmonary hemorrhage when injected intraperitoneally or intravenously in mice [ 30]. *In vivo* studies administering PLA_2_ from the Javan spitting cobra ( *Naja sputatrix*) intravenously and intratracheally in rats resulted in marked pulmonary inflammation and edema, supported by an increase in inflammatory markers and decreased protein expression of Na^+^/K^+^ ATPase and AQP [ 31]. Crotamine and crotoxin's high PLA_2_ activity also suggested evident pulmonary inflammation, edema, hemorrhage, and atelectasis in mice injected with the whole venom of the South American rattlesnake ( *Crotalus durissus*) intraperitoneally and intramuscularly [ 19, 32].

A human case report from Sri Lanka documented a lethal *Daboia russelli* bite that caused anaphylactic shock and significant renal failure, followed by pulmonary hemorrhage on day three [ 33]. The author hypothesized that the lung injury was rather caused by PLA_2_ in the venom than by a minor degree of hemolysis and coagulopathy. In India, one patient survived severe envenomation from a *Daboia russelli* but developed consumptive coagulopathy, acute renal failure, rhabdomyolysis, paralysis, and diffuse alveolar hemorrhage persisting a week after the bite. Plasmapheresis was proposed to aid the patient's recovery [ 34].

b) Cytotoxins: 

Most venoms amplify tissue damage synergistically along with PLA_2_ with cytotoxins. The toxins are highly basic, positively charged, amphipathic proteins that can create pores on the negatively charged cell membranes [ 35, 36]. Some types of cytotoxins, called cell-penetrating peptides (CPP), a family of short (less than 35 amino acids) naturally occurring or artificially produced peptides can also break cell membranes [ 37]. Some CPPs include melittin, anoplin, and mastoparans from wasps, latarcin from spiders, crotamine, crotalicidin, and elapid cathelicidin-related antimicrobial peptides from snakes, and pardaxin from fish skin [ 27].

Cytotoxins in terrestrial venomous animals (snakes, spiders, insects) and marine animals

Most cytotoxins belong to the three-finger toxin superfamily and are mostly found in cobra snake venom [ 35]. Other sources include sea anemones, cnidarians like multi-tentacled box jellyfish ( *Chironex fleckeri*), and Portuguese man o' war ( *Physalia physalis*) [ 6, 38]. Spider cytotoxin, such as phospholipase D (sphingomyelinase D) from recluse spiders ( *Loxosceles* spp.), can directly disrupt the alveolar cell membrane and indirectly lead to cytokine storms resulting in pulmonary edema [ 39, 40]. The presence of proteases, cytotoxins, and vasodilative peptides in stonefish venom (family Synanceja) caused lung edema, inflammation, and hemorrhage in animals and cardiogenic or non-cardiogenic pulmonary edema in human case reports [ 41, 42]. Jellyfish venom (genus *Nemopilema*) contains cytotoxins and metalloproteinases with pore-forming properties, which were suspected to cause cardiogenic shock and increase vascular permeability leading to fatal cases with pulmonary edema [ 43, 44]. Cantharidin found in blister beetles (genus *Epicauta*) has an acantholytic property and has been shown to cause cardiac injury, pulmonary edema, and subpleural hemorrhage in alpacas [ 45].

***Extracellular matrix (ECM) destruction***

The ECM area contains various connective tissues, including collagen, elastin, and proteoglycans [ 46], which provide mechanical and functional stability to capillaries and alveoli, making it a common target of exogenous toxins.

a) Matrix metalloproteinases (MMPs): 

The proteinase enzymes weaken capillary walls, collapse basement membranes, and promote the spread of toxins by hydrolyzing many structural proteins of the basal lamina component and surrounding ECM known as matrix metalloproteins [ 47]. These injuries increase the risk of pulmonary hemorrhage and contribute to inflammation by releasing numerous mediators present in alveolar exudates [ 47, 48]. Venom of jellyfish, cone snails, centipedes, scorpions, and snakes all contain large amounts of MMPs [ 6], such as snake venom MP (SVMP), which is often referred to as hemorrhagins due to its ability to cause bleeding.

MMPs in snake venom

SVMPs are ubiquitous in viperid venom [ 49]. Numerous animal studies have provided supporting evidence that SVMPs, such as Jararhagin, the main SVMP found in the venom of *Bothrops asper* and *Bothrops jararaca*, primarily target the basal lamina of alveolar cells, leading to pulmonary hemorrhage [ 50- 52]. The destructive effects on cellular structures can be further intensified by PLA_2_ [ 53, 54], another abundant enzyme in venom, as demonstrated by the increased detachment of endothelial cells when both enzymes are present [ 55]. Additionally, a C-type lectin (CTL) called aspercetin, which is another venom component, has been identified to potentiate the hemorrhagic effect [ 51]. 

MP activities in the venom of hump-nosed pit vipers ( *Hypnale* spp.) have also been implicated in inducing pulmonary edema and hemorrhage *in vivo* [ 56]. The venom of the Gaboon pit viper ( *Bitis gabonica*) has been shown to cause pulmonary edema in animal studies, possibly due to the venom's effect on the cardiovascular system or the hemorrhagic component damaging pulmonary endothelial cells [ 57, 58].

SVMP activities in venom have been implicated in pulmonary hemorrhage in several human case reports. A bite from *Bothrops jararacussu*, a pit viper with a high proportion of hemorrhaging, killed a 36-year-old woman within 45 minutes. Her autopsy revealed pulmonary hemorrhage and disseminated microthrombi in alveolar vessels [ 59]. Sri Lankan *Daboia russelli* venom, containing MP and other hemorrhagic toxins, was suspected of causing massive pulmonary hemorrhage in a 30-year-old man six hours after the bite, along with paralysis, renal failure, rhabdomyolysis, and deep vein thrombosis [ 60]. Pulmonary hemorrhage was also reported in patients bitten by *Hypnale hypnale*, accompanied by symptoms of thrombotic microangiopathy (TMA) such as renal failure, coagulopathy, and dry gangrene of both feet [ 61]. One fatal case from *H. hypnale* resulted in severe systemic bleeding, including intracerebral, endocardial, pericardial, and pulmonary hemorrhage and TMA. Despite aggressive resuscitation with blood products and hemodialysis, blood loss continued until death due to unavailable antivenom [ 62]. Autopsies of sudden death cases after *H. hypnale* bites have also revealed myocardial and pulmonary hemorrhage [ 63].

b) Hyaluronidase: 

Nearly all vertebrate cells contain hyaluronic acid, a negatively charged glycosaminoglycan that serves as an intercellular adhesive. Hyaluronidase damages local tissue by hydrolyzing lung interstitial hyaluronan, which inadvertently facilitates poison dispersal [ 64]. Hyaluronidases can be found in the secretions of nematodes and leeches, as well as in the venom of snakes, scorpions, centipedes, spiders, insects, fish, and lizards [ 64- 66]. In contrast to MMPs, animal studies or human case reports of lung injuries by hyaluronidase were not evident.

***Loss of cell-matrix adhesion***

Integrins mediate the adherence of eukaryotic cells to the ECM. On the alveolar surface, they function as extracellular receptors that regulate cell adhesion, proliferation, and migration to maintain alveolar homeostasis. Disintegrins and CTL derived from snake venom impair these integrin functions [ 24]. Animal-toxin disintegrins preferentially interact with certain types of alveolar integrins, particularly those that anchor to ECM collagens [ 67], and endothelial integrins, specifically vascular cell adhesion molecule-1 (VCAM-1) [ 24]. Disintegrins are generally not harmful (25), but dysfunctional integrins of alveolar epithelial cells might indirectly cause lung injury by triggering an inflammatory response [ 68].

## Impaired normal vascular function

***Toxins interfering with hemostasis***

Pulmonary hemorrhage from snake venom

Toxins, particularly those from snakes, can disrupt systemic hemostasis and lead to pulmonary hemorrhage [ 33, 50, 51, 59] and pulmonary embolism [ 69]. These toxins are capable of causing direct anti/prothrombotic effects, platelet dysfunction, indirect consumptive coagulopathy from secondary endothelial injuries, such as disseminated intravascular coagulation, thrombotic thrombocytopenic purpura, and hemolytic uremic syndrome [ 16], or enhancing other toxins hemorrhagic effects [ 51]. The detailed mechanisms of coagulopathy are reviewed elsewhere [ 70, 71]. The typical peptides/proteins involved in these events are as follows:

a) Proteases: 

Serine protease - These enzyme toxins affect different stages of blood coagulation and are frequently found in viperid snakes, spiders, and scorpions [ 72]. They can act either as pro-coagulants through fibrin synthesis, factor V activation, or platelet aggregation, or as anti-coagulants through fibrinolysis, plasminogen activation, or protein C activation [ 73].

MMP - Animal MMP has fibrinolytic properties and can proteolyze clotting proteins, which, in addition to injuring the BM, can cause pulmonary hemorrhage such as jararhagin found in *Bothrops jararaca* [ 71]. Similar to serine protease, several MMPs can promote thrombosis by activating prothrombin such as ecarin found in saw-scaled pit viper ( *Echis carinatus*) *,* and factor X such as RVV-X, a factor X activator, found in *Daboia russelli* [ 71]. 

b) Disintegrin and C-type lectin (CTL) toxins: 

Disintegrins, which can bind to platelet glycoprotein (GP) IIb/IIIa integrins, are cysteine-rich, Arg-Gly-Asp (RGD) - containing polypeptides identified in snake venom. Integrins are essential for the development of the platelet-platelet bridge and promote platelet aggregation. Because disintegrin is frequently present in complexes with metalloproteinase in SVMP classes P-II and P-III [ 74], it is understandable why snakebite victims have an increased risk of lung hemorrhage [ 73]. PIII-SVMP has a greater hemorrhagic potential due to the presence of a disintegrin-like domain that interferes with coagulation and a metalloproteinase domain that has a greater ability to hydrolyze type IV collagen and other non-fibrillar collagens in the BM-ECM network [ 48]. Like disintegrins, CTLs are also found in SVMP class P-III [ 74] and target platelet membrane integrins whose ligands include factor IX, factor X, or GPIb-mediated platelet activators [ 75]. 

c) PLA_2_: 

PLA_2_ especially those from viperids affects several blood coagulation processes by inhibiting the prothrombinase complex and interacting with numerous coagulation-related proteins and membranes [ 76].

d) L-amino acid oxidases (LAAOs): 

LAAOs are flavoenzymes that are present in a wide range of species, including bacteria, fungi, algae, fish, snails, and snakes (except for the Hydrophidae family) [ 77, 78]. They convert L-amino acid substrates into keto acids, ammonia, and hydrogen peroxide through oxidative deamination (H_2_O_2_). The production of H_2_O_2_ causes cytotoxicity to various cell types, including alveolar cells [ 77]. Additionally, LAAOs display both procoagulant activity by causing platelet aggregation and anticoagulant activity by weakening clots [ 79]. Intravenous injection of LAAO component in the pit viper *Agkistrodon blomhoffii ussurensis* venom to mice induced loss of pulmonary structure, pulmonary edema, and hemorrhage [ 77].

***Pulmonary embolism from snake venom***

While snake bites are commonly associated with hemorrhagic effects, there have also been reports of pulmonary embolisms in humans. A serine protease in *Bothrops jararacussu* (Jararacussin-1) has potentially contributed to the lethal bite from this snake by consumption of clotting factors through promoting weak fibrinogen clots [ 59]. *Bothrops lanceolatus*, a snake found only in Martinique, has been linked to thrombotic phenomena. A case series of 50 *B. lanceolatus* bites documented two incidences of pulmonary embolisms [ 80]. Another case report documented a fatal bite from *B. lanceolatus* that resulted in brain and myocardial infarction. A necropsy revealed a rupture of the papillary muscle of the mitral valve from a growing thrombus and the presence of numerous blood clots in the brain, lungs, mesentery, kidneys, and small arterial walls. Additionally, intense angiogenesis was noted in the organizing cerebral infarcts. The formation of these blood clots and abnormal angiogenesis could be induced by the presence of MMP and vascular endothelial growth factor (VEGF), respectively, in *B. lanceolatus* venom [ 81]. In another case involving *B. lanceolatus*, a woman on contraceptive pills suffered from serious pulmonary embolism and disseminated intravascular coagulation (DIC) [ 69].

Pulmonary embolisms can occur a few days after the snake bite and are often accompanied by DIC or hypofibrinogenemia. Excessive inflammation or direct toxin effects have been hypothesized as the underlying causes [ 82]. A delayed massive pulmonary embolism was observed in a case involving a Mojave rattlesnake ( *Crotalus scutulatus*) bite, occurring on day three despite receiving multiple doses of antivenoms [ 83]. Cases of viper bites in Morocco (most likely from *Vipera lebetina* or *Cerastes cerastes*) [ 84], Greece ( *Vipera ammodytes, V. aspis, V. lebetina,* or *V. xanthina)* [ 85] *,* and French western coast ( *Vipera aspis* or *V. berus*) [ 82] also exhibited delayed acute pulmonary embolism about one week after the bite.

***Toxins interfering pulmonary hemodynamics and vascular permeability***

Certain peptide toxins associated with inflammation can induce vasodilation, which increases vascular permeability and blood flow to the lungs [ 86]. Examples of such peptides include prostaglandins, histamines [ 87], and components of kallikrein-kinin pathways, such as bradykinins, kininogens, and kallikrein-like enzymes that increase bradykinin synthesis, as well as angiotensin-converting enzyme inhibitors that decrease bradykinin oxidation [ 86]. Most of these peptides can be found in the venom of insects, frogs, and reptiles [ 28, 87]. The toxins found in scorpion venoms have been frequently associated with pulmonary edema in human case reports [ 87]. Notably, a toxin discovered in the venom of the Indian red scorpion ( *Mesobuthus tamulus*) was named pulmonary edema-inducing toxin (PoTx) due to its capacity to cause lung injury *in vivo* [ 88].

## The inflammation 

Critical host response against envenomation entails innate and adaptive immune strategies aimed at venom detection, neutralization, detoxification, and symptom relief [ 2]. Venoms frequently offset the host's immune defense and produce even more severe symptoms. The principal causes of inflammation after envenomation include the following mechanisms.

***Tissue injury***

Activated toxin-injured vascular endothelium, whether local or distant from the site of injury, upregulates the expression of various mediators, including angiopoietin-2, and adhesion molecules such as intercellular adhesion molecule (ICAM), VCAM, and selectin. These mediators then attract and activate immune cells like neutrophils, macrophages, and lymphocytes [ 20]. Numerous proinflammatory cytokines, including tumor necrosis factor (TNF)-α, interferon (IFN)-γ, interleukin (IL)-8, IL-6, and IL-1β, are generated, drawing in additional immune cells [ 21]. Cell death follows membrane lipid peroxidation by H_2_O_2_, nitric oxide, and oxygen species produced by neutrophils [ 89]. Inflammation not only damages the endothelium layer but also weakens the tight junctions (vascular endothelial (VE)-cadherin) [ 20] and subtly disturbs the alveolar epithelium, which is more resilient to damage. This can result in even more excessive exudative fluid leaking into the alveolar septa or alveolar space [ 20]. The synthesis of mediators of inflammatory cells to the site of tissue damage, in turn, starts a vicious circle of inflammation [ 90]. Inflammation also favors local and systemic procoagulant states by activating platelets, and inhibiting tissue plasminogen, which may account for the presence of microthrombi in acute lung injury models [ 91].

**Figure 4.**
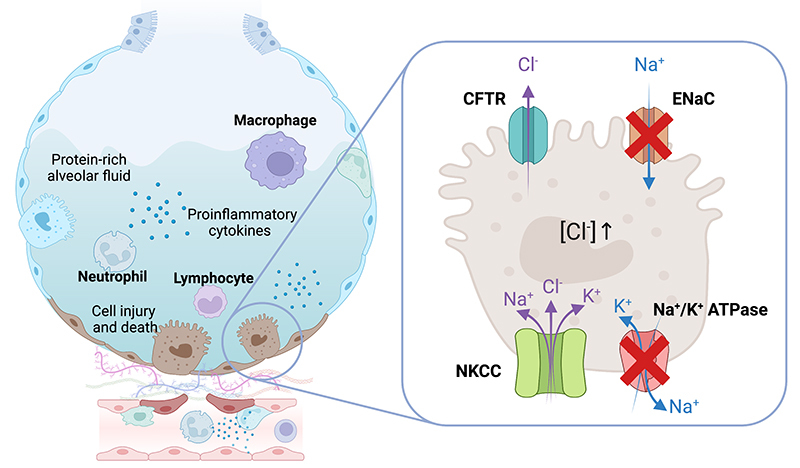
Schematic presentation of ion channels expressed (NKCC and CFTR) and inhibited (ENaC and Na^+^/K^+^ ATPase) on injured alveolar cells (both type I and type II) [21, 22]. Inflammation disrupts fluid balance through two main mechanisms: the release of vasodilators and the action of inflammatory mediators such as IL-1β, IL-8, and TNF-α. These mediators inhibit channels responsible for alveolar fluid clearance, including Na^+^/K^+^ ATPase, ENaC, and AQP5. Simultaneously, they increase the expression of NKCC and CFTR, which facilitate the inward transport of sodium and chloride-driven alveolar fluid secretion, respectively. CFTR: cystic #brosis transmembrane conductance regulator; ENaC: epithelial sodium channel; Na^+^/K^+^ ATPase: sodium/potassium ATPase pump; NKCC: Na^+^-K^+^-Cl^-^ cotransporter; Na^+^: sodium, K^+^: potassium, Cl^-^: chloride, H_2_O: water.

Inflammation dysregulates fluid homeostasis in the alveoli by the release of vasodilators such as prostaglandins, histamines, and nitric oxide from the wounded tissue [ 89]. Additionally, inflammatory mediators such as IL-1β, IL-8, and TNF-α inhibit channels responsible for alveolar fluid clearance including Na^+^/K^+^ ATPase, ENaC, CFTR, and AQP5 [ 21]. The mediators also increase expressions of Na^+^-K^+^-Cl^-^ cotransporter (NKCC) and CFTR (10, 90). Specifically, NKCC located on the basolateral side of alveolar cells facilitates inward transport of one molecule each of Na^+^ and K^+^ ions and two molecules of Cl^-^. The chloride molecules are then transported apically through CFTR (10) promoting chloride-driven alveolar fluid secretion ( Figure 4).

***Systemic inflammatory response syndrome***

In addition to directly injuring tissues, toxins produced by venomous animals can induce excessive inflammation by triggering a massive cytokine release. This phenomenon, known as a cytokine storm, can lead to a life-threatening condition called systemic inflammatory response syndrome (SIRS) [ 92] or cytokine release syndrome [ 93]. Symptoms and signs typically include fever [ 94], tachycardia, and tachypnea, as well as changes in immune cells populations such as leucocytes, and elevated levels of circulating acute phase reactants and cytokines, such as IL-1, IL-6, IFN-γ, and TNF-α [ 95]. If the inflammatory response outweighs the anti-inflammatory one and persists over time, multi-organ dysfunction may develop [ 96], including the aforementioned lung injury. Following envenomation, the histology of an alveolar epithelial cell revealed cellular senescence, polymorphonuclear cell infiltration, and fibrin deposition in the interstitial and alveolar spaces [ 19, 97, 98]. However, it is unclear whether the observed alveolar damage directly results from the venom components, an indirect effect mediated by inflammation, or both mechanisms [ 99] as most evidence comes from clinical observations and the detection of surrogate biomarkers for inflammation. However, the most commonly proposed mechanism is that certain toxins, particularly those found in scorpion venom, can disrupt the delicate balance of the neuroendocrine-immune axis (discussed further in 3.3). 

Cytokine storm by scorpions, snakes, insects, and spiders

Scorpion bites, notably of the genera *Tityus*, *Androctonus*, and *Buthus*, are widely recognized for their ability to trigger immune responses. [ 100]. Following toxin administration or bite, numerous *in vivo* studies and clinical cases have reported an elevation in proinflammatory cytokines [ 100] and clinical symptoms of SIRS which include severe outcomes such as cardiac and respiratory failure [ 101, 102]. Similar observations have been noted in *in vivo* studies and human case reviews involving venom from snakes of the genera *Crotalus* [ 98, 103], and *Bothrops* [ 104- 109]. Multiple bee stings, particularly from Africanized honeybees, and *Loxosceles* spider bites can also lead to a severe envenomation syndrome characterized by the significant release of cytokines as observed in SIRS [ 110, 111].

***Catecholamine excess***

Since electrical gradient plays a crucial role in cellular physiology and neuronal communication, many venoms contain neurotoxic peptides that promote or inhibit neurotransmission, typically through voltage-gated or ligand-binding ion channels such as Na^+^, K^+^, Ca^2+^, and Cl^-^ channels [ 112]. Some venomous animals, such as centipedes, spiders, scorpions, and snakes, also use the excruciating pain caused by their wounding apparatus to manipulate these channels [ 113].

Neuronal hyperexcitability, a state characterized by an increased level of endogenous monoamine neurotransmitters such as adrenaline, noradrenaline, acetylcholine, and dopamine, as well as other vasoactive peptides like neuropeptide-Y and endothelin-1, can lead to an "autonomic or catecholamine storm" [ 9]. These excessive catecholamines can induce the synthesis of pro-inflammatory mediators such as IL-6, IL-8, IL-10, and TNFs, resulting in a cytokine storm [ 114] that eventually contributes to pulmonary edema. This neuroendocrine-immune axis stimulation is frequently triggered by neurotoxic substances produced by scorpions (scorpion toxins - enhance Na^+^ and inhibit K^+^ and Cl^-^ channel) [ 9, 101], spiders (Black widow spider - *Latrodectus* spp., Funnel-web spider - *Atrax* spp.) (latrotoxin, atracotoxin - enhance Na^+^ and Ca^2+^ channel) [ 115, 116], and box jellyfish (Irukandji syndrome) (enhance Na^+^ channel) [ 117]. Pulmonary edema with evidence of increased sympathetic tone and inflammation is frequently reported in cases related to these toxins [ 10, 117, 118].

Catecholamine storm by scorpions, spiders, centipedes, and jellyfish

Scorpion venoms are abundant in neurotoxins that commonly induce adrenergic excess. The most clinically important toxins are α-toxins that inhibit the inactivation of neuronal Na^+^ channels [ 9], the reason behind pulmonary edema and hemorrhages (mostly fatal) in various human case reports [ 9, 10, 119, 120] and animal studies [ 121- 123]. Almost all these dangerous stings are by scorpions belonging to the family Buthidae - genera *Androctonus*, *Buthus*, *Tityus*, and *Mesobuthus*, and rarely, the family Caraboctonidae - genus *Hadruroides*. Similarly, neurotoxic α-latrotoxin and atracotoxin in venoms of spiders in the genus *Latrodectus* and *Atrax*, respectively, also cause pulmonary edema in humans by dysregulating the cardiovascular system during a catecholamine storm [ 116, 124, 125]. 

A centipede bite from the genus *Scolopendra* was reported to cause cardiogenic pulmonary edema in a 19-year-old female in India. Toxin-S and other vasoactive peptides in the venoms were believed to explain cardiac global hypokinesia with generalized ST depression and hypotension [ 126]. The mechanism of the cardiodepressant effect was still unclear, but the toxin's ability to dysregulate ion channels can induce severe vasospasm leading to heart failure *in vivo* [ 127].

Irukandji syndrome, characterized by severe catecholamine surge, is marine envenomation with similar features to human case reports to those of terrestrial habitats above [ 43, 117]. The syndrome is commonly caused by two families of the cubozoan jellyfish: Carukiidae (including the infamous *Carukia barnesi*) and Alatinidae [ 128].

***Hypersensitivity***

Venom-induced hypersensitivity

While envenomation-induced lung injury is marked by an exaggerated inflammatory response to venoms or bites and stings that can result in pulmonary edema, hemorrhage, and ARDS, another cause of inflammation-induced lung injury is venom-induced hypersensitivity [ 19, 32, 90, 98]. This type of injury is depicted by severe allergic reactions to venoms and can also lead to lung inflammation and injury. Type 1 hypersensitivities, characterized by IgE-mediated mast cell degranulation, are the most common allergic reactions. These reactions release pre-formed inflammatory mediators like histamine and proteases, leading to airway constriction and pulmonary edema [ 129]. The well-known allergens in animal toxins are those of hymenopteran (bee or wasp) venoms where PLA_2_, hyaluronidase, acid phosphatase, and dipeptidyl peptidase in honeybees, and PLA_1_ and antigen-5 in Vespa are the primary allergens [ 130]. Severe hymenopteran venom-induced anaphylaxis commonly causes pulmonary edema [ 2, 131, 132] and, rarely, pulmonary hemorrhage [ 133, 134]. The underlying processes may include consumptive coagulopathy from inflammation, IgE-mediated effects, or direct toxic venom (melittin) interference with the complement and bradykinin pathway [ 135, 136].

Antivenom-induced hypersensitivity

The hypersensitivity reaction is a common acute allergic complication of antivenom therapy, and evidence suggests that the reaction might result from complement activation, immunoglobulin complex, or antivenom impurities rather than being IgE-mediated [ 137]. Two cases of pulmonary edema were reported in India following the administration of polyvalent F(ab’)2 anti-snake venom (ASV). In Case 1, an 11-year-old child received ASV for mild local swelling after a cobra bite. After the first episode of mild allergic reactions (urticaria) subsided following adrenaline, antihistamine, and steroid, a rechallenge dose of antivenom was given. He developed hypotension and respiratory distress later confirmed to be caused by cardiogenic pulmonary edema as a side effect of the antivenom. The symptoms improved after a second dose of medications and mechanical ventilation [ 138]. In the second case, the patient developed severe anaphylaxis with pulmonary edema 90 minutes after ASV antivenom was given for a prolonged whole blood clotting test after a viperid bite. However, he fully recovered after supportive therapy [ 139].

## Neurologic Involvement: Respiratory Muscle Paralysis

The respiratory muscles play a key role in the lungs to maintain their functions. Many neurotoxins can cause respiratory muscle paralysis and acute respiratory failure. However, the explicit mechanisms of neurotoxins are beyond the purview of this article. Voltage-gated channels and neuromuscular junctions are the typical targets of neurotoxins. These neurotoxins and their targets are noteworthy to mention.

Toxins that affect neuromuscular junctions can be categorized into postsynaptic and presynaptic. Postsynaptic neurotoxins bind to nicotinic acetylcholine receptors and block the action of the neurotransmitter acetylcholine. Examples of these neurotoxins include α-cobratoxin which is present in cobras ( *Naja* spp.), α-bungarotoxin in kraits ( *Bungarus* spp.), 3FTxs α-neurotoxin in both black mamba ( *Dendroaspis polylepis*) and green mamba ( *D. angusticeps*) and acanthophin-D found in common death adder ( *Acanthophis antarcticus*) [ 140- 142]. Presynaptic neurotoxins bind to nerve terminals and block the release of acetylcholine. Examples of these neurotoxins are beta bungarotoxin found in kraits and P-elapitoxin-Aa1a in common death adder [ 140, 143, 144]. The victims of these snake bites typically experience muscle paralysis that progresses from small muscles to respiratory muscles and ultimately to total paralysis [ 145, 146]. 

Voltage-gated channels are located along nerve fibers and muscle cells. The opening/activation of Na^+^, and Cl^-^ channels and the closing of the K^+^ channel cause depolarization. Opening Ca^2+^ channels and depolarization activate the neurotransmitter release. While the contrary action of opening and closing these channels causes hyperpolarization and a decrease in neurotransmitter release [ 147]. Many toxins target voltage-gated sodium channels (VGSCs). There are 6 binding sites on VGSCs. The toxins that act on site 1 are tetrodotoxin, saxitoxin, and α-conotoxin. Tetrodotoxin can be found in many marine animals such as pufferfish ( *Tetraodon* spp.), blue-ringed octopus ( *Hapalochlaena lunulata*, *H. maculosa,* and *H. fasciata*), and horseshoe crab ( *Carcinoscorpius rotundicauda*) [ 148]. Saxitoxin which resembles tetrodotoxin in structure is found in freshwater pufferfish ( *Tetraodon fangi*) [ 149]. Alpha-conotoxins are found in some cone snails such as *Conus geographus, C. striatus,* and *C. textile* [ 150]. Victims will experience difficulty in limb control and paresthesia/anesthesia after consuming tetrodotoxin or saxitoxin-containing meals or being stabbed by cone snails. This could eventually lead to respiratory muscle paralysis and respiratory failure [ 151, 152]. 

## Overall Clinical Presentation and Therapy

Viperid snakebites are a leading cause of envenomation-induced lung injury, likely due to the larger size of these venomous animals and their specialized venom delivery system, which can release significant amounts of venom and cause serious clinical effects [ 153]. Other venomous terrestrial animals, such as scorpions, spiders, and centipedes, as well as marine species, especially jellyfish, can also cause such injuries. The presence of pulmonary manifestations, such as edema, hemorrhage, exudative infiltrate, embolism, and, in rare cases, bronchospasm, often indicates poor clinical outcomes. Table 1 summarizes the toxins, animal species, and characteristics of previous human case reports or *in vivo* evidence of toxin-induced lung injury. Respiratory signs and symptoms include dyspnea, shortness of breath, tachypnea, desaturation, hemoptysis, pink frothy sputum, wheezing, rales, and crepitations. In cases of severe hypoxia, tachycardia, bradycardia, hypertension, and altered consciousness may also be present [ 33, 97]. Although diffuse bilateral pulmonary infiltration is more common, asymmetrical, or unilateral pulmonary injuries have also been reported [ 160, 161].

**Table 1.** Exogenous (animal) toxins with evidence of directly causing lung injury in case reports.

Animals	Toxins	Pulmonary pathology				Mechanisms	Case	Ref.
		Hmrx	Edema	Inflam.	Throm.			
Snakes								
Viperids								
*Daboia russelli*	Phospholipase A_2_ (VRV-PL-VIIIa)	√				Alveolar cytotoxicity	Humans	[ 14, 18, 33, 60]
	MMP	√				Vascular damage		
	Procoagulant (cerebellar infarction)		√			Neurogenic pulmonary edema		
	Nephrotoxins		√			Renal failure		
*Hypnale hypnale*	Unknown hemorrhagic toxins	√				Unknown	Humans	[15, 61]
	Nephrotoxins		√			Renal failure		
*Crotalus* spp.	Whole venom (crotoxin, PLA_2_ crotamine)	√	√	√		Inflammation, vascular damage	Mice	[19, 32, 98]
*Crotalus scutulatus*	Unknown				√	Procoagulant state	Humans	[83]
*Agkistrodon blomhoffii ussurensis*	LAOO (ABU-LAO)	√	√				Mice	[77]
*Bothrops* spp.	Jararhagin (P-III SVMP)	√				Inflammation, vascular damage	Humans, mice	[50, 15, 59, 105, 106, 108]
	BaP1 (P-I SVMP)							
	Aspertin (CTL) in *Bothrops asper*							
	Jararacussin-1 (serine protease) in *Bothrops jararacussu*							
*Bothrops lanceolatus*	MMP				√	Procoagulant state	Humans	[69, 80, 81]
	VEGF							
*Vipera berus*	Unknown		√			Unknown	Humans	[154]
Vipers in Morocco, Greece, and French west coast	Unknown				√	Procoagulant state	Humans	[ 82, 84, 85]
*Bitis gabonica*	Hemorrhagin		√			Vascular damage	Animals (monkey, dogs, rats, guinea pigs, rabbit)	[57, 58]
	Nephrotoxins		√			Renal failure		
Elapids								
Most elapids	Neurotoxins		√			Respiratory paralysis	Humans	[16]
*Naja sputatrix*	Phospholipase A_2_		√	√		Inflammation (decreased fluid clearance)	Rats	[31]
*Pseudonaja textilis*	Cardiotoxins		√			Cardiotoxicity	Humans	[155]
*Bungarus* spp. (krait)	Neurotoxins, cardiotoxins		√			Cardiotoxicity, autonomic dysfunction	Humans	[156, 157]
**Toads (genus *Bufo*)**								
		√	√			Cardiodepressant (digitalis effect - inhibit Na^+^/K^+^ ATPase)	Humans, dogs	[13, 158]
Insects								
Hymenoptera	Phospholipase, Antigen-5, melittin	√	√			Inflammation, increased vascular permeability (Anaphylaxis)	Humans	[110, 131- 134]
Blister beetle (genus *Epicauta*)	Cantharidin	√	√			Acantholysis	Alpacas	[45]
Spiders								
*Loxosceles* spp.	Phospholipase D (Sphingomyelinase D)		√			Inflammation, alveolar cytotoxicity	Humans, mice	[39, 111]
*Latrodectus* spp.	α-latrotoxin		√			Catecholamine storm	Humans	[116]
*Atrax* spp.	Atrachotoxin		√			Catecholamine storm	Humans	[124, 125]
Scorpions								
	Vasoactive peptides	√	√	√		Inflammation	Humans, rats	[9, 87, 88, 96, 102]
	PoTx (*Mesobuthus tumulus*)					Catecholamine storm		
	Neurotoxin (ex. α-toxin)							
**Centipede (*Scolopendra* spp.)**								
	Toxin S					Cardiotoxicity	Humans, animals	[126]
	Vasoactive peptide					Increased vascular permeability		
Jellyfish								
*Nemopilema nomurai*	Unspecified		√			Cytotoxicity (pore forming)	Humans, animals	[44]
*Chiropsalmus quadrumanus* and *C.quadrigatus*	Unspecified		√			Cardiotoxicity	Humans	[159, 163]
*Chironex fleckeri*	Unspecified		√			Cardiotoxicity	Humans	[164]
Irukandji jellyfish (ex. *Carukia* spp.)	Unspecified		√			Irukandji syndrome	Humans	[117]
Stonefish (family Synaceia)								
	Unspecified	√	√	√		Inflammation Alveolar cytotoxicity	Humans, animals	[41]
Sea anemone								
*Actinia equina*	Equinatoxin II		√			Increased vascular permeability	Animals	[165]

Abbreviation: Hmrx - hemorrhage; Inflam. - inflammation; LAOO - L-amino acid oxidases; MMP - matrix metalloproteinase; N/A - not available; PoTx - pulmonary edema-producing toxin; RVBCMP - Russell’s viper basic coagulant metalloproteinase; SVMP - snake venom metalloproteinase; Throm. - thrombosis; VEGF - vascular endothelial growth factor; VRV-PL-VIIIa - *Vipera russelli*venom phospholipase A_2_ fraction VIIIa

Lung damage occurs nearly immediately at the cellular level [ 19], but clinical signs may be immediate (within minutes to hours) or delayed (days) depending on the mechanisms. Although anaphylaxis occurs acutely (a few minutes), significant sequelae such as pulmonary edema and, less frequently, bleeding, can take longer (a few hours) [ 131- 134]. Prothrombotic actions may take a few hours to cause pulmonary embolism [ 69]. Pulmonary edema or ARDS from sympathetic overactivity is more immediate (minutes to hours) when compared to the process of excessive inflammation and subsequent multiorgan failure, which may take days [ 44, 97, 117, 126, 162]. Pulmonary hemorrhage can occur at any time from a few hours [ 30, 50, 59, 60] to many days, and is often accompanied by systemic coagulopathy [ 33, 62].

The main treatment for envenomation-induced lung injury is still airway and breathing support therapy. Adrenaline, steroids, and antihistamines are specifically used to treat pulmonary edema or bronchospasm caused by anaphylaxis. Only antivenoms are used as specific antidotes in human case reports. Blood components and antivenoms are frequently provided to patients who experience pulmonary hemorrhage, with varying degrees of success [ 33, 60, 62]. Supporting the primary target organ is the main goal of treatment for pulmonary edema caused by cardiotoxins or neurotoxins.

## Conclusion

Animal toxins can inflict severe damage to the respiratory system of the host, resulting in various pulmonary manifestations, including but not limited to edema, hemorrhage, or embolism. These serious complications of envenomation require prompt and effective management, as they have the potential to result in unfavorable clinical outcomes. Therefore, understanding the mechanisms underlying toxin-induced lung injury and developing efficacious treatment modalities are imperative for enhancing patient outcomes and reducing the associated mortality rates linked to envenomation.
